# Correction: Prolonged mitosis results in structurally aberrant and over-elongated centrioles

**DOI:** 10.1083/jcb.20191001906192020c

**Published:** 2020-06-23

**Authors:** Dong Kong, Natalie Sahabandu, Catherine Sullenberger, Alejandra Vásquez-Limeta, Delgermaa Luvsanjav, Kimberly Lukasik, Jadranka Loncarek

Vol. 219, No. 6 | 10.1083/jcb.201910019 | April 9, 2020

The authors noticed that an incorrect image had been inadvertently included in the paper. The middle centriole with a cilium in Fig. 1 E is the same electronmicrograph that had been previously used to illustrate a cilium in Sahabandu et al. (2019. *Journal of Microscopy*. https://doi.org/10.1111/jmi.12841), Fig. 3 I. This panel has been replaced with the correct centriole image; the revised Fig. 1 is shown below. All versions of the article have been corrected. This error appears only in PDF versions downloaded on or before June 23, 2020.

